# Drought stress had a predominant effect over heat stress on three tomato cultivars subjected to combined stress

**DOI:** 10.1186/s12870-017-0974-x

**Published:** 2017-01-25

**Authors:** Rong Zhou, Xiaqing Yu, Carl-Otto Ottosen, Eva Rosenqvist, Liping Zhao, Yinlei Wang, Wengui Yu, Tongmin Zhao, Zhen Wu

**Affiliations:** 1 Institute of Vegetable Crop, Jiangsu Academy of Agricultural Science, Nanjing, Jiangsu China; 2 Jiangsu Key Laboratory for Horticultural Crop Genetic Improvement, Nanjing, Jiangsu China; 3 College of Horticulture, Nanjing Agricultural University, Nanjing, Jiangsu China; 4 Department of Food Science, Aarhus University, Årslev, Denmark; 5 Department of Plant and Environmental Sciences, University of Copenhagen, Taastrup, Denmark

**Keywords:** Tomato, Drought stress, Heat stress, Photosynthesis, Chlorophyll fluorescence

## Abstract

**Background:**

Abiotic stresses due to environmental factors could adversely affect the growth and development of crops. Among the abiotic stresses, drought and heat stress are two critical threats to crop growth and sustainable agriculture worldwide. Considering global climate change, incidence of combined drought and heat stress is likely to increase. The aim of this study was to shed light on plant growth performance and leaf physiology of three tomatoes cultivars (‘Arvento’, ‘LA1994’ and ‘LA2093’) under control, drought, heat and combined stress.

**Results:**

Shoot fresh and dry weight, leaf area and relative water content of all cultivars significantly decreased under drought and combined stress as compared to control. The net photosynthesis and starch content were significantly lower under drought and combined stress than control in the three cultivars. Stomata and pore length of the three cultivars significantly decreased under drought and combined stress as compared to control. The tomato ‘Arvento’ was more affected by heat stress than ‘LA1994’ and ‘LA2093’ due to significant decreases in shoot dry weight, chlorophyll a and carotenoid content, starch content and NPQ (non-photochemical quenching) only in ‘Arvento’ under heat treatment. By comparison, the two heat-tolerant tomatoes were more affected by drought stress compared to ‘Arvento’ as shown by small stomatal and pore area, decreased sucrose content, Φ_PSII_ (quantum yield of photosystem II), ETR (electron transport rate) and q_L_ (fraction of open PSII centers) in ‘LA1994’ and ‘LA2093’. The three cultivars showed similar response when subjected to the combination of drought and heat stress as shown by most physiological parameters, even though only ‘LA1994’ and ‘LA2093’ showed decreased F_v_/F_m_ (maximum potential quantum efficiency of photosystem II), Φ_PSII_, ETR and q_L_ under combined stress.

**Conclusions:**

The cultivars differing in heat sensitivity did not show difference in the combined stress sensitivity, indicating that selection for tomatoes with combined stress tolerance might not be correlated with the single stress tolerance. In this study, drought stress had a predominant effect on tomato over heat stress, which explained why simultaneous application of heat and drought revealed similar physiological responses to the drought stress. These results will uncover the difference and linkage between the physiological response of tomatoes to drought, heat and combined stress and be important for the selection and breeding of tolerant tomato cultivars under single and combine stress.

## Background

Abiotic stresses caused by environmental factors could adversely affect the growth and development of crops [1]. Crops respond to the abiotic stresses with various modifications on morphological, cellular, physiological, biochemical and molecular level [2–4]. In the last decade, lots of studies focused on the response of crops to a single stress [3, 5, 6]. However, several abiotic stresses usually occur concurrently and crops are always subjected to a combination of different abiotic stresses in the field [1, 7]. Among the abiotic stresses, drought and heat stress are two critical threats to crop growth and sustainable agriculture worldwide [8–10]. Drought stress as a consequence of insufficient rainfall or deficient soil moisture might induce various biochemical, physiological and genetic responses in plants, which severely restricted crop growth [11, 12]. Heat stress due to global warming has increasingly deleterious effect on crop production and heat stress is quite common for the crops cultivated through summer [13]. Heat stress is frequently associated with drought stress in field conditions [14], which makes necessary the study of crops responding to combined heat and drought stress.

Tomato (*Solanum lycopersicum* L., formerly *Lycopersicon esculentum* Mill.) is one of the most important vegetables in the world. Many studies have described the physiological response of tomatoes to drought and heat stress applied individually [4, 15–18]. The combined effect of drought and heat has been mainly studied in chickpea [10], tobacco [19], *Arabidopsis* [20, 21] and wheat [2, 22]. Response of plants to combined drought and heat could not be directly extrapolated from the response of plants to the individual stresses [19–21, 23]. Tomato often encounter the combination of drought and heat stress during its cultivation. However, effect of the combined drought and heat stress on tomato and the relationship between the physiological responses of tomatoes to single and combined stress remained unclear.

Compared to individual stress, combined stress of drought and heat induced the expression of *HSP*s in wheat [22] and induced specific proteins in wild barley [24]. There could be different responsive mechanism of plants to combined stress with individual stress [7, 21, 23]. Under drought stress, inhibition of plant photosynthesis by stomatal closure caused the unbalance between light reaction and Calvin-Benson cycle as a consequence of limited CO_2_ diffusion into the leaf [25]. By comparison, heat stress restrained plant photosynthesis mainly through affecting biochemical reactions [26, 27]. Heat tolerant wheat cultivars could maintain high rates of photosynthesis and stomatal conductance during heat stress, while decreased rates of photosynthesis induced decreased stomatal conductance in sensitive cultivars [28]. Photosynthetic products, namely sugars, specifically accumulated during the combination of drought and heat stress [20].

Photosystem II (PSII) is a sensitive component to heat stress [29]. Chlorophyll fluorescence is an efficient and non-destructive technique to measure the photochemical efficiency of PSII and thereby detect the damage of stress in PSII [30]. The maximum potential quantum efficiency of PSII (F_v_/F_m_) provides an estimate of the maximum quantum efficiency of PSII, which is primarily affected by heat stress [4, 31].

The aim was to (i) dissect growth performance and physiological responses of tomatoes under drought, heat and combined stress in terms of leaf relative water content, pigment content, stomatal characteristics, photosynthesis, carbohydrate content and chlorophyll fluorescence; (ii) compare the tolerance of three tomato cultivars to combined stress. We hypothesized that (1) the tomatoes showed heat tolerance might not show the combined stress tolerance; (2) combined drought and heat stress might cause specific response on tomatoes than single stress, or similar response to single stress when one of the single stress played a predominant role on tomato. This study will help us to uncover the difference and linkage between the physiological response of tomatoes to drought, heat and combined stress and provide us theoretical basis for improving tomato tolerance to more instable climates.

## Methods

### Plant material and growth condition

One common greenhouse tomato cultivar ‘Arvento’ (*Solanum lycopersicum*) (Rijk Zwaan Nederland B.V, De Lier, Netherlands) and two heat-tolerant tomatoes based on our previous research including ‘LA1994’ (*S. lycopersicum*) and ‘LA2093’ (*S. pimpinellifolium)* (Tomato genetics resource centre, TGRC, University of California, CA, USA) were used [4]. Seeds were sown in a plug tray. After 18 days, the seedlings were transferred to plastic pots (11-cm diameter, 9 cm height) with commercial sphagnum substrate (Pindstrup 2; Pindstrup Mosebrug A/S, Ryomgaard, Denmark). The seedlings were grown in a greenhouse under long-day conditions with supplementary light provided by SON-T lamps (Phillips, Eindhoven, Netherlands). The average daytime photosynthetic photon flux density (PPFD) was 250 − 350 μmol m^–2^ s^–1^ in the greenhouse with 60 ± 5% relative humidity (RH) and 400 ppm CO_2_. Air temperature was 24 ± 3 °C during the day and 18 ± 3 °C during the night. The seedlings were irrigated by flooding the benches every morning for 10 min with a full nutrient solution (PH 6.0, EC 2.34 mS cm^-1^, N 185 mg L^-1^, P 27 mg L^-1^, K 171 mg L^-1^, Mg 20 mg L^-1^ and full micro nutrients).

In order to reduce the complexity of interactions between genetic and environmental effects on phenotype, the study was conducted in climate chamber, where the onset of some abiotic stresses can be clearly defined, such as increasing the temperature and withdrawing the irrigation without the influence of rainfall. The 21-day-old uniformly sized seedlings were transferred to climate chambers (MB teknik, Brøndby, Denmark). Environmental parameter settings of the chambers were 26/20 °C for day/night, 60% RH, 400 ppm CO_2_, a 14-h photoperiod with 300 ± 20 μmol m^–2^ s^–1^ PPFD. The seedlings were irrigated with the full nutrient solution once every day for 10 min. The 25-day-old seedlings were randomly divided into four groups with 12 plants per cultivar for each treatment. The experiment included four treatments: (1) control, 26/20 °C (day/night) with three times of irrigation every day; (2) heat stress, 32/26 °C (day/night) with three times of irrigation every day; (3) drought stress, 26/20 °C (day/night) without irrigation; (4) combination of heat and drought stress, 32/26 °C (day/night) without irrigation. The treatments lasted for five days since the tomatoes under single drought and combine stress showed significant phenotype change such as leaf wilting on day 4, which become severe on day 5 during the preliminary experiment.

### Destructive harvest and leaf temperature

Plant height and internode length was measured with a ruler on day 4. Leaf number was counted and leaf angle between the leaf and main stem was measured with a protractor on day 4. The plants were harvested on day 5. Leaf area was immediately measured with a leaf area meter (model 3100, LI-COR, Lincoln, Nebraska, USA) after the harvest. Shoot fresh weight (above cotyledonary node) was measured by cutting the seedling from the cotyledonary node. The shoot dry weight was measured after drying the shoot in an oven at 85 °C for 48 h. There were four replications from four plants per cultivar and per treatment for the measurements of plant height, internode length, leaf number, leaf angle, leaf area and shoot fresh and dry weight.

In situ leaf temperature of four plants per cultivar and per treatment was measured with a Raynger 3i infrared gun (Raytek, Santa Cruz, CA, USA) during the 10th hours of the light period on day 2 and day 4. Average leaf temperature of the two days was the final result.

### Relative water content

One leaflet from the first fully expanded leaf of four plants per cultivar and per treatment was cut from a plant on day 5. Fresh weight (FW) of the leaflet was immediately measured after cutting. Then, the leaflet was immersed in dd-H_2_O in a petri dish and incubated under normal room temperature. After four hours, the leaflet was taken out, properly wiped to remove the water on the surface of the blade and weighed to obtain turgid weight (TW). Afterwards, the leaflet was put in a drying oven for 24 h and weighed to obtain dry weight (DW). Relative water content (RWC in %) = [(FW - DW)/(TW - DW)] * 100.

### Chlorophyll content

Pigment content was determined using the method of Wintermans and De Mots (1965) [32]. Two leaf disks (1.54 cm^2^ for each) were punched out from the first fully expanded leaf of four plants per cultivar and per treatment without the main vein using a cork borer, immersed in 10 mL 95% cold ethanol and incubated in 4 °C in darkness for 48 h. The absorption was measured at 470, 649 and 665 nm using a UV-VIS spectrophotometer (Shimadzu UV-1700, Kyoto, Japan). Chlorophyll a (Chl a), chlorophyll b (Chl b) and carotenoid (Car) contents per unit area (mg/cm^2^) were calculated by the formula Chl a = (13.95 × A_665_ − 6.88 × A_649_)/1.54, Chl b = (24.96 × A_649_ − 7.32 × A_665_)/1.54 and Car = (10^3^ × A_470_ − 2.05 × Chl a − 114.8 × Chl b)/1.54, respectively.

### Stomatal anatomy

Abaxial side of the first fully expanded leaf without the main vein was used for impression of stomata with elite HD+ (Zhermack, Badia Polesine, Italy) using the method of Giday et al. [33]. Four leaflets from four plants per cultivar and per treatment were sampled on day 5. Six non-overlapping fields of each sample were taken pictures using a magnification of 19.2×. Stomatal density was accessed on 24 fields per treatment. The length, width and area of stomata and pore were measured on 48 randomly chosen stomata per treatment (two stomata per field).

### Gas exchange and carbohydrate content

The temperature response curve for net photosynthetic rate (*P*_N_), intracellular CO_2_ concentration (*C*_i_), stomatal conductance (*g*_s_) and transpiration rate (*E*) was measured using a portable photosynthesis system (CIRAS-2, PP Systems, Amesbury, USA). Measurement of four plants per cultivar and per treatment started day 4 at a temperature of 24 °C followed by 26 °C, 28 °C, 30 °C, 32 °C, 34 °C and 36 °C. Light intensity was 300 μmol m^–2^ s^–1^ provided by LED light source (PP Systems, Amesbury, USA). The first fully expanded leaf was placed in situ in a cuvette (1.7 cm^2^) and the measurements were recorded every 10 s until *P*_N_ and *g*_s_ reached a steady state. Average of the last five values was used as the final result for each temperature level. To maintain the vapor pressure deficit (VPD) at 1.0-3.4 kPa, a moist cloth was placed on the water vapor equilibrator of the CIRAS-2 when the VPD was above 2.0 kPa; the air was dehumidified when the VPD was below 0.8 kPa.

During the last 2 h of the light period on day 5, the first fully expanded leaf was harvested, immediately frozen in liquid nitrogen and stored at −80 °C. The samples of four plants per cultivar and per treatment were freeze-dried (Gamma 1-20, LMC-1, Struers., Denmark) for three days, ground in a mixer mill (MM200, Retsch Inc., Haan, Germany) and weighed. The content of glucose, fructose, sucrose and starch was extracted and measured as described by Zhou et al. [4].

### Chlorophyll fluorescence

Chlorophyll fluorescence measurement was performed on the first fully expanded leaf of four plants per cultivar and per treatment on day 4. For quenching analysis, the seedling was dark adapted for 20 min in a dark room. The F_v_/F_m_ was measured with a MINI-PAM (Walz, Effeltrich, Germany) operated with the WinControl software. Φ_PSII_ = F_q_′/F_m_′ (quantum yield of PSII), q_L_ (fraction of open PSII centers), NPQ (non-photochemical quenching) and ETR (electron transport rate) [30] were measured under a PPFD of 300 μmol m^-2^ s^-1^ with external light source. Actinic light was provided by a halogen lamp (Schott KL 1500, Göttingen, Germany) through fiber optics. Light level was manually controlled. A micro quantum and thermo sensor on the leaf clip holder recorded the incident PPFD and leaf temperature.

### Data analysis

Analysis of variance (ANOVA) between the parameters of plants under control, drought, heat and drought + heat stress of heat and drought were performed using SPSS 16.0 (SPSS Inc. Chicago, IL, USA).

## Results

### Plant growth and leaf relative water content

**Fig. 1:**
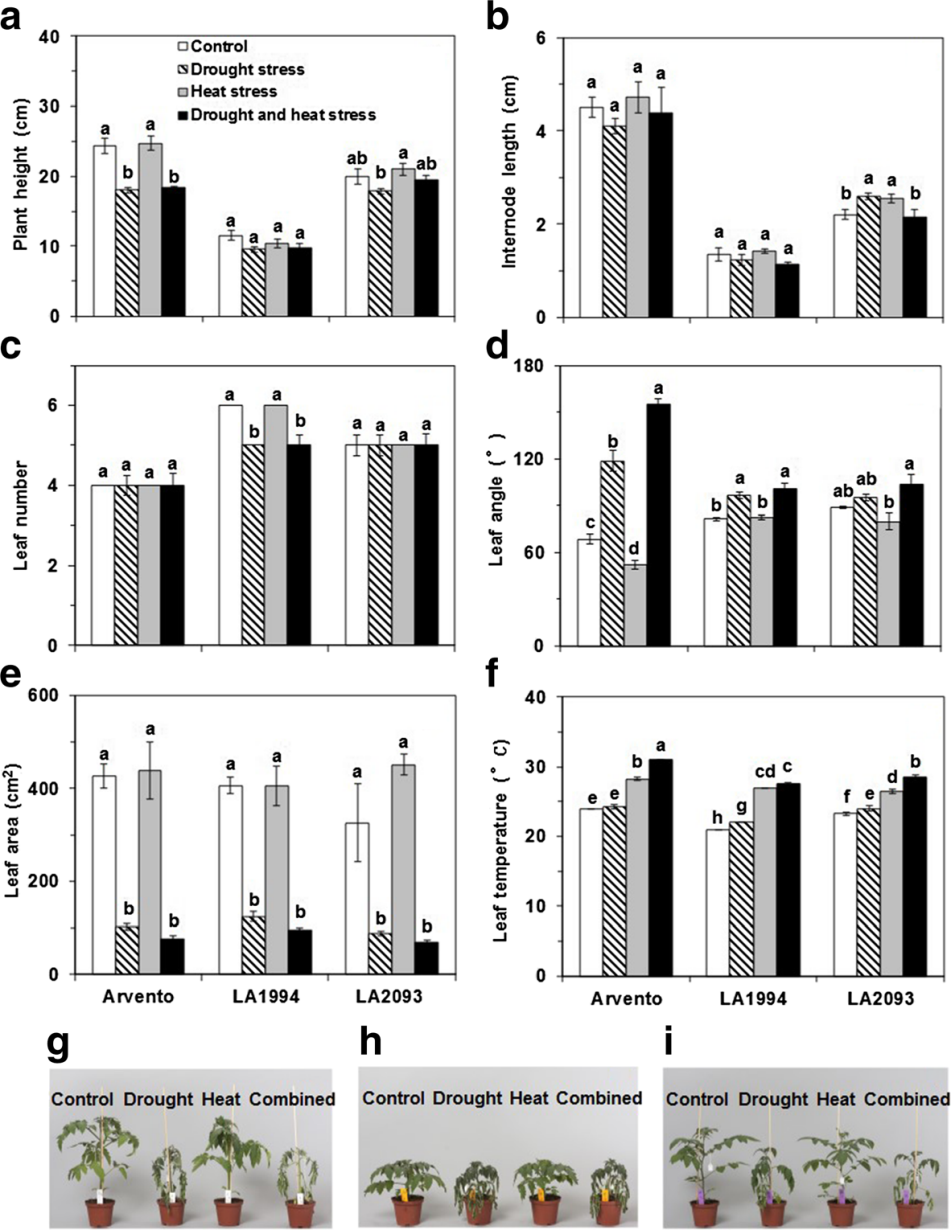
Plant height and leaf growth parameters of the three tomato cultivars under four days of control, drought, heat and combined stress. Different sub-graphs represent **a** plant height, **b** internode length, **c** leaf number, **d** leaf angle, **e** leaf area, **f** leaf temperature of the three tomato cultivars and phenotypes of **g** ‘Arvento’, **h** ‘LA1994’ and **i** ‘LA2093’ under control, drought, heat and combined stress. The data represent mean values ± SE (*n* = 4). Different small letters above the bars indicate significant differences (*P* < 0.05)

Plant height of ‘Arvento’ significantly decreased under drought stress and combined stress compared to control (Fig. 1a). The plant height of ‘LA2093’ was significantly lower under drought stress than heat stress (Fig. 1a). Combined stress had no significant effect on the plant height of ‘LA1994’, being the shortest of the three cultivars in all four treatments (Fig. 1a). Individual drought and heat stress significantly increased the internode length of ‘LA2093’ (Fig. 1b). The leaf number of ‘LA1994’ was significantly lower under drought stress and combined stress than control (Fig. 1c). The leaf angle of ‘Arvento’ significantly decreased under heat stress in comparison with control (Fig. 1d). In contrast to this the leaf angle of ‘Arvento’ and ‘LA1994’ significantly increased under drought and combined stress compared to control (Fig. 1d). For ‘LA2093’, the leaf angle of plants was significantly smaller under heat stress than combined stress (Fig. 1d). The leaf area was significantly lower under drought and combined stress than control (Fig. 1e). The leaf temperature of ‘Arvento’ was significantly higher during heat and combined stress than control and drought stress (Fig. 1f). For ‘LA1994’ and ‘LA2093’, the leaf temperatures of plants were significantly higher under stresses than control (Fig. 1f). In all cultivars, plants under drought and combined stress showed wilted stem and leaf and similar phenotype, while plants under heat stress did not show apparent damage compared to the control (Fig. 1g, h, i).

**Fig. 2:**
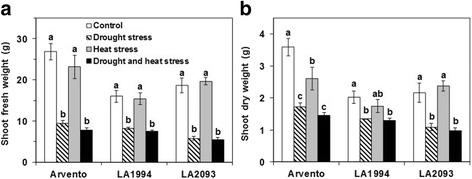
**a** Shoot fresh weight and **b** shoot dry weight of the three tomato cultivars under four days of control, drought, heat and combined stress. The data represent mean values ± SE (*n* = 4). Different small letters above the bars indicate significant differences (*P* < 0.05)

The shoot fresh weight of the three cultivars significantly decreased under drought and combined stress compared to control (Fig. 2a). The shoot dry weight of ‘Arvento’ significantly decreased under stresses (Fig. 2b). For ‘LA1994’ and ‘LA2093’, the shoot dry weight of plants was significantly smaller under drought and combined stress than control, while it was unaffected by heat stress alone (Fig. 2b).

**Fig. 3:**
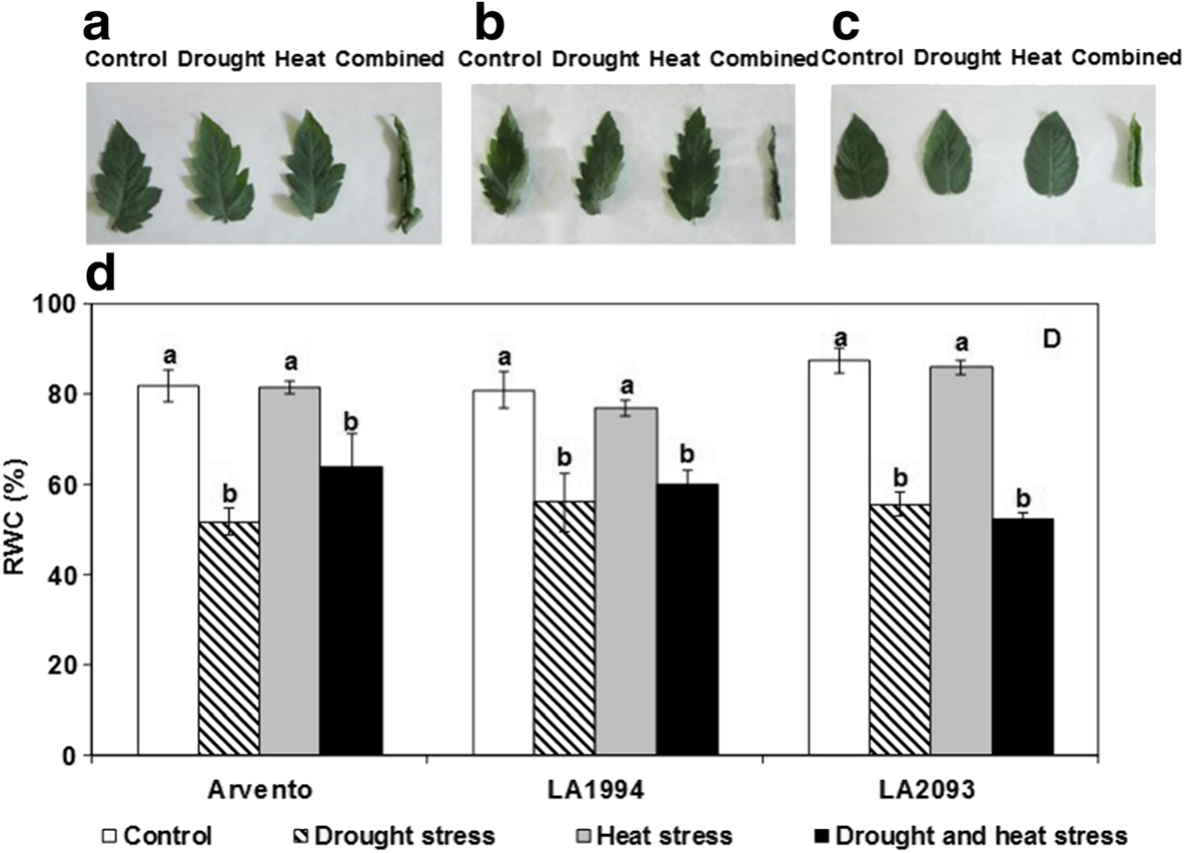
Relative water content (RWC, %) of the three tomato cultivars under four days of control, drought, heat and combined stress. Different sub-graphs represent leaflets after being immersed with ddH_2_O from **a** ‘Arvento’, **b** ‘LA1994’ and **c** ‘LA2093’ and **d** RWC of the three tomatoes under control, drought, heat and combined stress. The data represent mean values ± SE (*n* = 4). Different small letters above the bars indicate significant differences (*P* < 0.05)

The leaflets of plants under control and individual stresses recovered, while the leaflets under combined stress stayed wilted after being immersed with ddH_2_O (Fig. 3a, b, c). The relative water content of all cultivars significantly decreased under drought and combined stress in comparison with control, while it was unaffected by heat alone (Fig. 3d).

### Pigment content

**Fig. 4:**
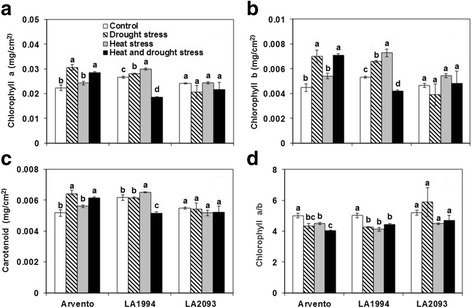
Leaf pigment content per unit area of the three tomato cultivars under four days of control, drought, heat and combined stress. Different sub-graphs represent **a** chlorophyll a, **b** chlorophyll b, **c** carotenoid and **d** chlorophyll a/b. The data represent mean values ± SE (*n* = 4). Different small letters above the bars indicate significant differences (*P* < 0.05)

The Chl a and Chl b content in ‘Arvento’ significantly increased under drought and combined stress, while in ‘LA1994’ individual and combined stress significantly increased and decreased Chl a content, respectively (Fig. 4a, b). The change of Car content in ‘Arvento’ was the same as that of Chl a and Chl b content (Fig. 4c). The individual heat stress and combined stress significantly increased and decreased the Car content in leaves of ‘LA2093’, respectively (Fig. 4c). Chl a/b of leaves from ‘Arvento’ and ‘LA1994’ significantly decreased under single and combined stress in comparison with control (Fig. 4d). The chlorophyll composition was unaffected by the treatments in ‘LA2093’ (Fig. 4a, b, d).

### Stomatal characteristics

**Fig. 5:**
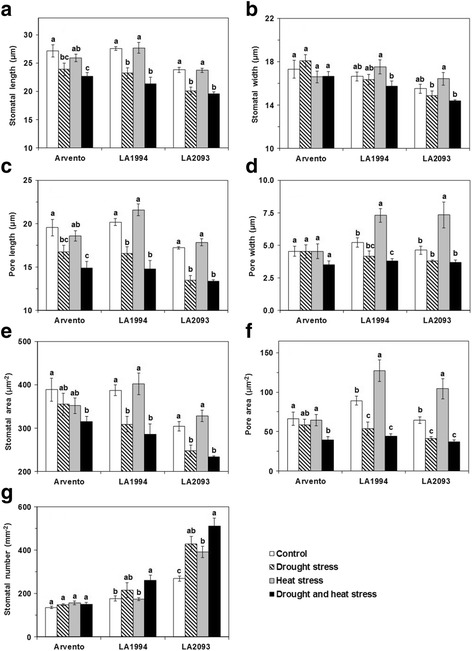
Stomatal characteristics in the abaxial leaves of the three tomato cultivars under four days of control, drought, heat and combined stress. Different sub-graphs represent **a** stomatal length, **b** stomatal width, **c** pore length, **d** pore width, **e** stomatal area, **f** pore area and **g** stomatal number. The data represent mean values ± SE (*n* = 4). Different small letters above the bars indicate significant differences (*P* < 0.05)

The stomatal and pore length significantly decreased in all cultivars under drought and combined stress compared to control (Fig. 5a, c), while the stomatal width was unaffected by any stress (Fig. 5b). The pore width of ‘LA1994’ and ‘LA2093’ significantly increased under heat stress, while it decreased in ‘LA1994’ under the combined stress in comparison with control (Fig. 5d). Stomatal and pore area was significantly smaller than the control in ‘Arvento’ at combined stress and ‘LA1994’ and ‘LA2093’ under drought and combined stress (Fig. 5e, f). The pore area significantly increased under heat stress in ‘LA1994’ and ‘LA2093’ compared to control (Fig. 5f). The stomatal number was significantly higher than control under combined stress in ‘LA1994’ and under all stress treatments in ‘LA2093’ (Fig. 5g).

### Temperature response curves of gas exchange and carbohydrate content

**Fig. 6:**
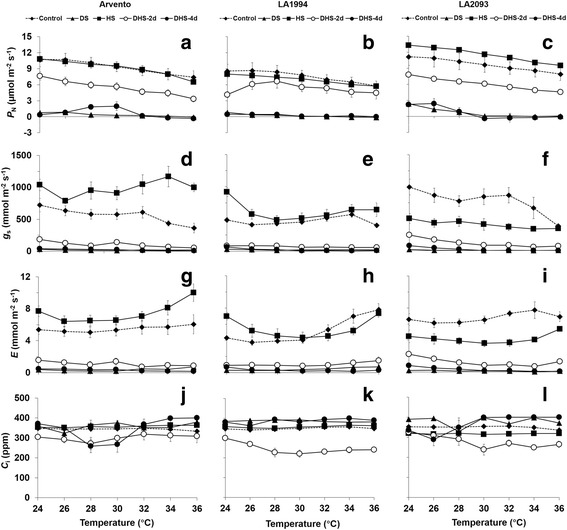
Temperature response curve in the leaves of the three tomato cultivars under control, drought, heat and combined stress on day 4 and for the combined stress also on day 2. Different sub-graphs represent **a**, **b**, **c** net photosynthetic rate (*P*_N_), **d**, **e**, **f** stomatal conductance (*g*_s_), **g**, **h**, **i** transpiration rate (*E*) and **j**, **k**, **l** intracellular CO_2_ concentration (*C*_i_). The data represent mean values ± SE (*n* = 4)

The *P*_N_ of ‘Arvento’ and ‘LA2093’ under combined stress significantly decreased already on day 2 compared to control irrespective of temperature, while the heat stress was not different from the control on day 4 (Fig. 6a, c). In the drought and combined stress treatments, *P*_N_ dropped to almost zero at most temperatures after four days in all three cultivars (Fig. 6a, b, c), which was accompanied with almost zero *g*_*s*_ (Fig. 6d, e, f) and *E* (Fig. 6g, h, i). The *g*_s_ of ‘Arvento’ and ‘LA2093’ under heat stress were respectively higher and lower than control at most temperatures, (Fig. 6d, f), while it was mostly unaffected in ‘LA1994’ (Fig. 6e). The *E* of all cultivars followed the same patterns as *g*_*s*_ (Fig. 6g, h, i).

**Fig. 7:**
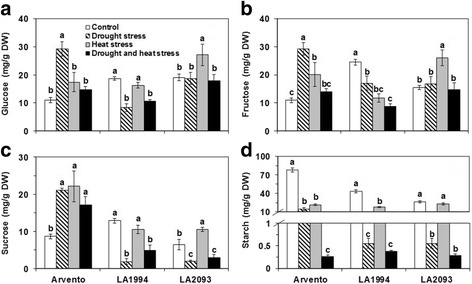
Carbohydrates content in the leaves of the three tomato cultivars under four days of control, drought, heat and combined stress. Different sub-graphs represent **a** glucose, **b** fructose, **c** sucrose and **d** starch. The data represent mean values ± SE (*n* = 4). Different small letters above the bars indicate significant differences (*P* < 0.05)

The glucose content of ‘Arvento’ was significantly higher under drought stress than control, while the glucose content significantly decreased in ‘LA1994’ under drought and combined stress and significantly increased in ‘LA2093’ under heat stress (Fig. 7a). The fructose content was significantly higher than control in ‘Arvento’ under individual drought and heat stress and in ‘LA2093’ under heat stress, but fructose content in ‘LA1994’ under all stresses was significantly lower than control (Fig. 7b). Compared to control, the sucrose content of ‘Arvento’ significantly increased under all stresses, but significantly decreased in ‘LA1994’ and ‘LA2093’ under drought and combined stress (Fig. 7c). The sucrose content of ‘LA2093’ was significantly higher under heat stress than control (Fig. 7c). The starch content significantly decreased in three cultivars under all stresses compared to control, except for ‘LA2093’ under heat stress (Fig. 7d).

### Chlorophyll fluorescence

**Fig. 8:**
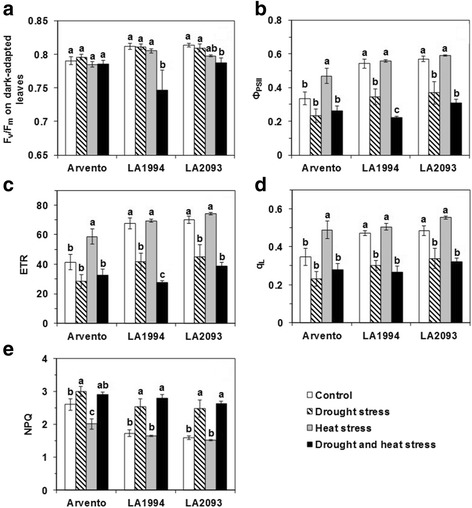
Chlorophyll fluorescence measured at a PPFD of 300 μmol m^-2^ s^-1^ at room temperature in the three tomato cultivars under four days of control, drought, heat and combined stress. Different sub-graphs represent **a** F_v_/F_m_ on dark-adapted leaves, **b** quantum efficiency of PSII (Φ_PSII_), **c** electron transport rate (ETR), **d** fraction of open PSII centers (q_L_) and **e** non-photochemical quenching (NPQ). The data represent mean values ± SE (*n* = 4). Different small letters above the bars indicate significant differences (*P* < 0.05)

The F_v_/F_m_ of ‘LA1994’ and ‘LA2093’ was significantly lower under combined stress than control (Fig. 8a). Compared to control, Φ_PSII_, ETR and q_L_ of ‘Arvento’ significantly increased under heat stress, while they significantly decreased in ‘LA1994’ and ‘LA2093’ under drought and combined stress (Fig. 8b, c, d). NPQ significantly increased in ‘Arvento’ under drought stress but decreased under heat stress in comparison with control (Fig. 8e). For ‘LA1994’ and ‘LA2093’, NPQ significantly increased under drought and combined stress (Fig. 8e).

## Discussion

Drought and heat stress has severely restricted world crop production, which has caused a big loss for agricultural economy [22, 34, 35]. The response of tomato plants to single drought or heat stress have been previously described extensively [4, 15–18].

When drought and heat was applied in combination, the response of plants could not be compared to the conclusions drawn from individual stress experiments for tobacco [19] and *Arabidopsis* [20, 21]. Compared to control, the internode length of plants of ‘LA2093’ grown under combined stress was not affected, while it increased under individual drought and heat treatment. Similarly, the leaf angle of ‘Arvento’ under combined drought and heat stress was larger than control, while the leaf angle of the plants under individual drought and heat stress increased and decreased, respectively. These results partly confirmed that the effect of combined drought and heat stress to tomato differ from individual stress. This might be due to different, sometimes even opposing, signaling pathways induced by combined stress [7].

Combined drought and heat stress usually create more severe damage than individual stresses in a temperate annual/biannual plant community [36] and in crops such as barley [37] and chickpea [10]. The combination of drought and heat stress have potential negative interaction [1, 7, 38]. Barley at 36/32 °C (day/night) for seven days with the soil water content being 15% field capacity and chickpea at 32:20 °C (maximum: minimum temperature) with 50% leaf water content were used to study the combined effect of drought and heat stress [10, 37]. Even though emerging evidence show that plant responses to combined stresses are unique, plants exhibit shared responses which are common to individual stresses and stress combination [38]. Moreover, the combined stress does not always increase the damage on plants than single stress as shown in this study. The combination effect of drought and heat stress could be similar to single stress when one of the single stress played a predominant role on tomato. We found that heat stress did not damage the three tomato cultivars as most of the leaf physiological parameters showed no difference between tomatoes under control and heat stress, which was in accordance with the results of Nankishore and Farrell [39]. In contrast to the expected negative effect of stress combination on tomato growth, the combination of heat and salinity showed a remarkable protection level to tomatoes from salinity stress [23]. Similarly, drought stress played predominant role as indicated by significant damage on tomatoes caused by drought rather than heat. As a consequence, most of the responses of the three tomatoes including leaf area, shoot fresh and dry weight, leaf relative water content (RWC), stomatal and pore length and temperature response curve under combined stress were similar to single drought stress. As shown by Vile et al., [40], no specific trait was affected only by the combination of heat and drought among the traits investigated in *Arabidopsis*. Similarly, we found that the main responses caused by simultaneous occurrence of drought and heat stress were caused by single drought stress in tomatoes. For example, leaf area, shoot fresh and dry weight of all three cultivars under combined stress was similar to the drought treatment, and significantly lower than under control and heat treatment. The alteration of these three growth indexes coincided with the change of *P*_N_ due to the interconnection between growth/yield and photosynthesis [23]. There was independency between the mechanisms of plants in responsive to drought and heat stress in *Arabidopsis* [40]. Drought stress has been shown to inhibit photosynthesis mainly by the closure of stomata due to cellular water depletion and subsequent reduction of assimilate transport [25], which was also seen in our experiment. In contrast, heat stress affecting the *P*_N_ through the biochemical reactions of photosynthesis [26–28]. The drought treatment caused a considerably larger inhibition of *P*_N_ than the heat treatment in all three cultivars due to lower *g*_s_. Therefore, drought and heat stress limit tomato photosynthesis and might ultimately decrease tomato yield. Drought and the combination of drought and heat stress caused >80% reduction in photosynthesis in tobacco plants, while heat stress did not decrease photosynthesis [19], which was similar with our conclusion in tomatoes. In case of combined heat and drought stress differing in severity, the physiological responses of tomatoes are apparently determined by the most severe stress in this case drought stress, as concluded by Pandey et al. [38]. Drought stress caused a greater effect than heat stress on yield and seed-filling in chickpea [10]. Thus drought stress might cause more severe reduction in tomato yield than heat stress as a consequence of the decreased *P*_N_, leaf area, shoot fresh and dry weight. However, the effect of single and combined stress on tomato flowering, fruit set and yield need further research.

Although the three cultivars maintained high *P*_N_ during heat stress, the stomatal regulation differed. ‘Arvento’ had higher *g*_s_, ‘LA1994’ was unaffected while ‘LA2093’ showed generally a lower *g*_s_ during heat stress. While neither ‘Arvento’ nor ‘LA1994’ showed a decrease in *C*_i_ during heat stress, the low g_s_ in ‘LA2093’ decreased *C*_i_ even though a high *P*_N_ could be maintained. As soon as drought was one of the stresses, *g*_s_ drastically decreased. A decrease in *P*_N_ could be the consequence of stomatal limitation where a decreased *g*_s_ was accompanied by declining *C*_i_ or non-stomatal factors with no difference in *C*_i_ [41]. After two days of combined heat and drought stress, *P*_N_ decreased due to stomatal limitation in all three cultivars. After four days of single drought or combined stress, however, *P*_N_ was reduced to almost zero due to biochemical limitations since *C*_i_ was unaffected at most temperatures, despite *g*_s_ < 50 mmol m^-2^ s^-1^. The only deviation was in ‘Arvento’ at 28-30 °C and ‘LA2093’ at 24-28 °C, where low *P*_N_ was still maintained after four days of combined stress, as reflected in a lowering of *C*_i_. The *P*_N_ of tomatoes under control, heat stress for four days and combined stress for two days decreased with increased temperature, indicating that the high temperature adversely affected the *P*_N_ of tomatoes, when there was no stress or the stress did not completely restrict the *P*_N_. Sustained high *P*_N_ in heat tolerant cultivars required high *g*_s_, which allowed high *E* and better cooling of the leaves than heat susceptible cultivars in wheat [28] and tomato [4]. We found that the cultivars differing in heat sensitivity did not show difference in the sensitivity of combined stress, which indicated that selection for one type of tolerance might not improve the tolerance to combined stress.

Sucrose, the primary end product of photosynthesis, is translocated from source leaf to sink organ through the phloem [42]. Sucrose content in ‘LA1994’ and ‘LA2093’ under single drought and combined stress decreased as compared to control, which might be due to lower *P*_N_ and reduced photosynthetic gain. However, sucrose in ‘Arvento’ leaf under stress accumulated, probably as sugar production rate in leaf exceed the export rate due to insufficient sink activity [43]. This can in turn lead to a decrease in *P*_N_ in ‘Arvento’ as a consequence of feedback inhibition and attenuate the source-link balance [43]. *Arabidopsis* subjected to combination of drought and heat stress accumulated sucrose that played a role as the major osmoprotectant [20], indicating that sucrose might play function as osmoprotectant in ‘Arvento’ under stress and sucrose content could be an important selection indicator for plants treated under drought and heat stress. Another indicator could be starch content, since only ‘Arvento’ had lower starch content under single heat stress as compared to control. Besides, the starch content of ‘Arvento’ were significantly higher than ‘LA1994’ and ‘LA2093’ under single drought stress. These indicated that ‘Arvento’ had better drought tolerance than ‘LA1994’ and ‘LA2093’, while ‘LA1994’ and ‘LA2093’ had better heat tolerance than ‘Arvento’. Combined stress tolerance in maize was distinct from individual stress tolerance in maize [44]. In contrast, the three tomato genotypes showed identical tolerance pattern to the combined stresses even though they showed different tolerance to single stress in this study. Single drought and combined stress induced premature senescence in the photosynthetic source leaf as indicated by wilting leaf, low *P*_N_ and reduced shoot weight. This might be followed by reducing the number and growth of the harvestable sink fruit through affecting the transport of assimilate [45] to avoid the feedback inhibition of leaf photosynthesis by coordinating the transport of assimilate between source leaf and fruit sink, which might delay leaf senescence [46].

Chlorophyll content strongly depends on the species’ physiological responses and their ability to tolerate stress [3]. Chlorophyll content is one of the most effective indicators for heat and drought tolerance identification of tomatoes [39]. High temperature could increase the chlorophyll content of heat-tolerant tomato [4]. Drought stress caused an increase in chlorophyll content in the upper leaves of tobacco plants, while the stress accelerated senescence in the lower leaves [47]. These explained why the chlorophyll content of the first fully expanded leaf increased in ‘LA1994’ under heat stress and in ‘Arvento’ and ‘LA1994’ under drought stress. In a previous study, the heat-tolerant tomato line had a higher stomatal number, bigger stomatal and pore size and a relatively lower leaf temperature under heat stress than control due to increased cooling capacity [4]. Accordingly, heat treatment induced bigger pore width and area in two heat-tolerant tomatoes in this study. However, stomatal closure is one of the first steps in response to drought stress and it relates to dehydration avoidance by reducing water loss and maintaining water status during unfavorable conditions [48]. This explained the decreased length and area of stomata and pore under individual drought and combined drought and heat stress.

Leaf relative water content (RWC) was established as an indicator of water status balance and plants with different leaf RWC could indicate a corresponding difference in leaf hydration, leaf water deficit and physiological water status [3, 49]. Decrease in RWC was a main factor causing decreased growth responding to osmotic stress in plants [50]. Under drought stress, sensitive pea genotypes were more affected by the decline in RWC than tolerant genotypes [51]. However, in our study, RWC decreased but with no significant difference between the tomato cultivars under drought and combined stress. This suggested that the three cultivars had similar sensitivity when subjected to drought and combined stress.

Short-term drought did not affect the efficiency of primary photochemical processes of PSII or the associated F_v_/F_m_ [52]. Photorespiration steps in the sink for ATP and NADPH protect PSII from damage when *g*_s_ decreases [30], which explained why F_v_/F_m_ of three cultivars was not affected by drought treatment. Therefore, the F_v_/F_m_ in vivo is not an effective parameter for distinguishing tomato genotypic variation under drought stress even though the F_v_/F_m_ in vitro was effective in a previous study [39]. Noteworthy, in this study, combined drought and heat treatment decreased the F_v_/F_m_ of two heat-tolerant tomatoes even though the F_v_/F_m_ of two heat-tolerant tomatoes under individual stress was not affected. This proved that the combination of heat and drought could aggravate the damage to tomatoes to some extent in accordance with the conclusion from Dreesen et al. [36] and Rollins et al. [37]. Moreover, we noticed a significant decrease in Φ_PSII_, ETR, q_L_ and increase in NPQ of two heat-tolerant tomatoes under drought and combined stress. This indicated that the severe stomatal closure after four days of drought related stress reduced the CO_2_ supply to chloroplasts [53]. Not only did P_N_ of the heat-tolerant tomatoes decrease, but also ETR, which was compensated by increased heat dissipation through NPQ protecting PSII from stress induced photoinhibition in drought stress alone, while the combined drought and heat stress was severe enough to damage PSII as indicated by the lower F_v_/F_m_ in the two cultivars. By contrast, chlorophyll fluorescence parameters except NPQ was not affected in ‘Arvento’ under drought and combined stress.

## Conclusions

This study was designed to clarify the physiological response of tomato plants to combined drought and heat stress, two of the most common abiotic stresses that usually act together. The tomato ‘LA1994’ and ‘LA2093’ could tolerate heat stress better than ‘Arvento’ as indicated by significant decreases in shoot dry weight, chlorophyll a, carotenoid and starch content and NPQ only in ‘Arvento’ under heat treatment. By contrast, the two heat-tolerant tomatoes were more affected by drought stress compared to ‘Arvento’ as indicated by small stomatal area and pore area, decreased sucrose content, Φ_PSII_, ETR and q_L_ in ‘LA1994’ and ‘LA2093’ under drought treatment. Even though only ‘LA1994’ and ‘LA2093’ showed decreased F_v_/F_m_, Φ_PSII_, ETR and q_L_ under combined stress, the three tomato cultivars showed similar response when subjected to the combination of drought and heat stress as shown by other physiological parameters. The cultivars differing in heat sensitivity did not show difference in the combined stress sensitivity, which indicated that selection for one type of tolerance might not improve the combined stress tolerance. Simultaneous application of heat and drought to the three tomato cultivars reveals similar responses mainly in plant growth, photosynthesis and chlorophyll fluorescence to the application of single drought stress. Therefore, the responses of tomato to combined heat and drought are predominantly controlled by water limitation instead of heat stress in this study. However, the effect of stress especially for combined stress on tomato fruit setting and yield need further studies.

## Abbreviations

Car: Carotenoid
Chl a: Chlorophyll a
Chl b: Chlorophyll b
*C*_i_: Intracellular CO_2_ concentration
DW: Dry weight
*E*: Transpiration rate
ETR: Electron transport rate
F_v_/F_m_: Maximum potential quantum efficiency of photosystem II
FW: Fresh weight
*g*_s_: Stomatal conductance
NPQ: Non-photochemical quenching
*P*_N_: Net photosynthetic rate
PPFD: Photosynthetic photon flux density
PSII: Photosystem II
q_L_: Fraction of open PSII centers
RH: Relative humidity
TW: Turgid weight
VPD: Vapor pressure deficit
Φ_PSII_: Quantum yield of photosystem II
